# T178. PRIOR SUB-THRESHOLD PSYCHOTIC SYMPTOMS ASSOCIATED WITH THICKER RIGHT INFERIOR FRONTAL GYRUS AMONG PATIENTS IN A FIRST EPISODE OF PSYCHOSIS

**DOI:** 10.1093/schbul/sby016.454

**Published:** 2018-04-01

**Authors:** Rachel Rosengard, Carolina Makowski, Jai Shah, Ashok Malla, Ridha Joober, Martin Lepage

**Affiliations:** 1 McGill University; 2 McGill University, Douglas Mental Health University Institute

## Abstract

**Background:**

Individuals with attenuated or sub-threshold psychotic symptoms (STPS) are considered at-risk for psychosis. The notion that STPS represent “early psychosis” holds promise as it suggests the possibility of charting the developmental course of psychotic illness with neuroimaging. However, recent evidence suggests that a significant minority of patients in a first episode of psychosis (FEP) do not recall pre-onset STPS, suggesting diversity in early positive symptom course. This diversity may be reflected at the level of neurodevelopment. While imaging studies of at-risk youth and FEP patients reveal progressive trends in cortical thinning across stages of illness, none have considered the STPS history of FEP patients. To better understand neurobiological trends across illness stages, we investigate the relationships between STPS history and cortical thickness in FEP patients using a whole-brain approach.

**Methods:**

Patients (N=93) were recruited from a specialized early intervention clinic for FEP at the Douglas Mental Health University Institute. The Circumstances of Onset and Relapse Schedule was administered to identify youth who recalled at least one of nine expert-selected STPS prior to their FEP (STPS+, N=67) compared to those who did not (STPS-, N=26). These STPS include: Suspiciousness or odd ideas of reference, odd or bizarre ideas that are not delusional, unusual or eccentric behavior, unusual perceptual experiences that are not clearly psychotic, disorganized or odd speech, inappropriate affect, hallucinations or delusions (sub-threshold), and passivity experiences. Age and sex-matched healthy controls were recruited (N=83) for comparison. Participants were scanned on a 1.5T MRI scanner between 1 and 3 times at baseline, at 1-year follow-up, and at 2-year follow-up. Structural T1-weighted images were processed through the CIVET 2.1 pipeline. Cortical thickness values of 320 scans (143 HC, 123 STPS+, 54 STPS-) that passed quality control were extracted for group analysis. Linear mixed effects models controlling for effects of age, sex, education, and mean whole-brain thickness were applied to obtain vertex-wise F-test maps comparing groups.

**Results:**

Post-hoc vertex-wise t-test maps were thresholded with Random Field Theory (p-cluster=0.001) and revealed that compared to controls, only STPS- patients exhibited significantly thinner cortical thickness in the right inferior frontal gyrus (peak t(162.3)=4.13, p<0.001). Examination of mean cortical thickness within this cluster, comparing patient groups only, revealed that compared to STPS+ patients, STPS- patients exhibited significantly thinner cortical thickness (t(172)=-2.55, p=0.01). This difference was most pronounced at baseline.

**Discussion:**

These results indicate that within the right prefrontal cortex, STPS+/- patients may undergo different cortical maturation trajectories leading up to and through a first episode of psychosis. These differences may explain differential vulnerability to sub-threshold psychotic symptomology before a full-blown episode. In addition to suggesting differential underlying neurobiology related to STPS, these results suggest the importance of considering STPS history in mapping the trajectories of cortical thickness among FEP patients.

